# Dual-factor Synergistically Activated ESIPT-based Probe: Differential Fluorescence Signals to Simultaneously Detect α-Naphthyl Acetate and Acid α-Naphthyl Acetate Esterase

**DOI:** 10.1021/acs.analchem.1c02945

**Published:** 2021-10-25

**Authors:** Kui Wang, Beidou Feng, Yonggang Yang, Yuehua Chen, Yuzhu Wang, Yafu Wang, Lin Yang, Kai Jiang, Tony D. James, Hua Zhang

**Affiliations:** † Key Laboratory of Green Chemical Media and Reactions, Ministry of Education; Henan Key Laboratory of Organic Functional Molecule and Drug Innovation; School of Chemistry and Chemical Engineering; Henan Normal University, Xinxiang 453007, P. R. China; ‡ Department of Hepatobiliary and Pancreatic Surgery, Henan Provincial People’s Hospital, Zhengzhou 450003, P. R. China; § Department of Chemistry, University of Bath, Bath BA2 7AY, U.K.

## Abstract

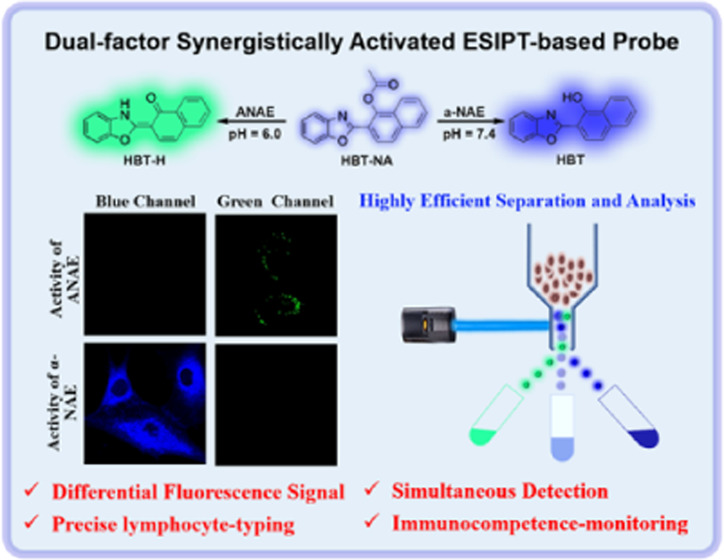

α-Naphthyl acetate esterase (**α-NAE**) and acid α-naphthyl acetate esterase (**ANAE**), a class of special esterases, are important for lymphocyte typing and immunocompetence-monitoring. As such, the simultaneous detection of **α-NAE** and **ANAE** has become a target to effectively improve the accuracy in lymphocyte typing. Therefore, we developed a dual-factor synergistically activated ESIPT-based probe (**HBT-NA**) to detect **α-NAE** and **ANAE** sensitively, rapidly, and simultaneously in a differential manner. **HBT-NA** exhibits differential fluorescence signal outputs toward small changes of **α-NAE** and **ANAE** activities. **HBT-NA** displays a weak fluorescence signal at 392 nm over a pH range from 6.0 to 7.4. However, when it interacts with **α-NAE** (0–25 U) at pH = 7.4, the fluorescence intensity at 392 nm enhanced linearly within 60 s (F_392 nm_/F0_392 nm_ = 0.042 C_**α-NAE**_ + 1.1, *R*^2^ = 0.99). Furthermore, **HBT-NA** emits ratiometric fluorescence signals (F_505 nm_/F_392 nm_) for **ANAE** (0–25 U) at pH = 6.0 within 2.0 min, exhibiting a good linear relationship (F_505 nm_/F_392 nm_ = 0.83C_**ANAE**_ – 1.75, *R*^2^ = 0.99). The differential fluorescence signals can be used to simultaneously detect the activities of **α-NAE** and **ANAE** in solutions and complex living organisms. More importantly, based on the differential fluorescence signals toward **α-NAE** and **ANAE**, T lymphocytes and B lymphocytes could be successfully typed and differentiated among nontyped lymphocytes, facilitating the real-time evaluation of their immune functions using flow cytometry. Hence, **HBT-NA** could be used for the ultrasensitive detection of the enzyme activities of **α-NAE** and **ANAE**, the real-time precise typing of lymphocytes, and the monitoring of immunocompetence.

α-Naphthyl acetate esterase (**α-NAE**) and acid α-naphthyl acetate esterase (**ANAE**) are two of the typical nonspecific esterases. Like other nonspecific esterases, **α-NAE** and **ANAE** exhibit a catalytic hydrolytic function for short-chain fatty acids.^1,2^ That is, they can catalyze the hydrolysis reaction of naphthyl acetate derivatives to generate α-naphthol by breaking down the acetic acid ester bond in living organisms.^3^ Although they belong to the nonspecific esterases, **α-NAE** and **ANAE** exhibit unique roles in the field of cell biology and medical diagnosis due to their catalytic hydrolytic functional characteristics.^4^ For example, **α-NAE** is a marker for leukemia diagnosis, typing and prognosis, and also for myeloid leukemia cell differentiation,^5^ while **ANAE** could be used to distinguish T lymphocytes that have cellular immunity function from B lymphocytes with humoral immunity function. T lymphocytes directly attack invaders and release cytokines that can then activate other parts of the immune system, while B lymphocytes produce antibody molecules that can latch on and destroy the invading viruses or bacteria.^6^ But, unlike other nonspecific esterases, the catalytic hydrolytic function of **α-NAE** and **ANAE** can only be activated by the action of two factors, that is, the enzymatic activity (biological species) and an appropriate pH (environmental conditions).^1,2^**α-NAE** must be at neutral pH (approximately pH 7.4), while **ANAE** must be at acid pH (approximately pH = 5.9–6.3). Many investigations have unequivocally demonstrated that such differences of pH conditions are key factors for activating their catalytic hydrolytic function. Importantly, such differences can affect their roles in the field of cell biology and medical diagnosis.^7^ More importantly, such differences in the pH of activation provide an approach for the differential detection of **α-NAE** and **ANAE**. Thus, to that end, the rapid and highly sensitive recognition output signals that are specifically regulated by two factors (*i.e*., the enzymatic activity and appropriate pH conditions) become important challenges to be overcome for the simultaneous and differential detection of **α-NAE** and **ANAE**.

In clinical diagnosis, the azo salt staining method is a gold standard for the detection of **α-NAE** and **ANAE** for serum analysis and cell staining. Significantly, the sensitivity of the method is relatively low. Therefore, a better method for the detection of **α-NAE** and **ANAE** is required. Toward that goal, excited-state intramolecular proton transfer (ESIPT) probes are a potential solution to that problem. Since ESIPT probes exhibit unique optical and physical properties, such as two output signals, rapid proton transfer, emission band with large stokes shift, unique four-level photochemical process, and so on, they have resulted in important fluorescence-based tools for analytical chemistry, molecular logic gates, and luminescent materials.^8−10^ Recently, ESIPT-based probes have been developed to monitor biomolecules or biomolecular events in a living organism, especially proteins and enzymes.^11−13^ Due to the extremely rapid proton transfer speed (*k*_ESIPT_ > 10^12^ s^–1^), a simple and effective 2-(2′-hydroxyphenyl) benzoxazole derivative (**HBT**)-based ESIPT probe was developed for the detection of ONOO^–^which exhibits good selectivity and a fast response time.^14^ Based on the transient nature of the four-level photochemical process and irreversible chemical reaction, a **HBT** ESIPT fluorophore, where the hydroxyl group has been protected by a tertbutyldiphenylchlorosilane, exhibited high sensitivity at the ppb level for fluoride.^15^ However, a **HBT** cyanine probe was designed to exhibit a large stokes shift 234 nm when activated at pH 5, which can effectively avoid undesirable inner-filter and/or self-reabsorption effects.^16,17^ These reported provide some guidance for design strategies toward ESIPT-based probes.^14,18^ In addition, a number of fluorescent probes combining ESIPT with AIE have been reported that overcome some of the inherent problems associated with ESIPT-based systems.^19−21^ Unfortunately, the application of many reported ESIPT-based probes are limited under certain circumstances (such as the detection of high-fidelity signals), since they are easily affected by environmental factors and, as such, generate off-target fluorescence changes.^22^ In addition, the fluorescence output signals of these probes can only be regulated by a single factor and are therefore unable to accurately monitor species that are regulated by multiple factors.^23^ Moreover, they are not suitable for the simultaneous and differential monitoring of multiple biological species, for example, the catalytic hydrolytic function of **α-NAE** and **ANAE**.

With this research, the hydrolysis reaction of naphthyl acetate derivatives was selected as the specific recognition reaction. Considering their catalytic hydrolytic function that is affected by the two factors, pH and the enzymatic activity, **α-NAE** and **ANAE** were selected as biomarker targets for the design of a synergistically activated ESIPT-based probe. Therefore **HBT-NA** was developed, where the fluorogen is 2-(benzo[*d*]oxazol-2-yl)phenol. In the absence of **α-NAE** or **ANAE**, **HBT-NA** emitted a weak blue fluorescence signal at 392 nm. But in the presence of α**-NAE** and **ANAE**, **HBT-NA** can emit differential fluorescence signals at different wavelengths to generate both ratiometric and off–on responses. We anticipated that the differential fluorescence signals of **HBT-NA** could be used to detect **α-NAE** and **ANAE** sensitively and specifically in cells simultaneously. Given the molecules’ differential fluorescence signals toward **α-NAE** and **ANAE**, we anticipated that **HBT-NA** could accurately type T lymphocytes and B lymphocytes and simultaneously evaluate their immunocompetence.

## Experimental Section

### Chemicals and Materials

The solvents and reagents used in this work for molecular synthesis and purification were of analytical grade. The solvents and reagents used in this work for molecular characterization were chromatographic grade. In the colocalization analysis, two commercial dyes were purchased from Thermo Fisher Scientific Company. LysoTracker Red, a commercial dye, was for the lysosome, and 5(6)-CFDA, a commercial dye, was for the cytoplasm.

**Scheme 1:**
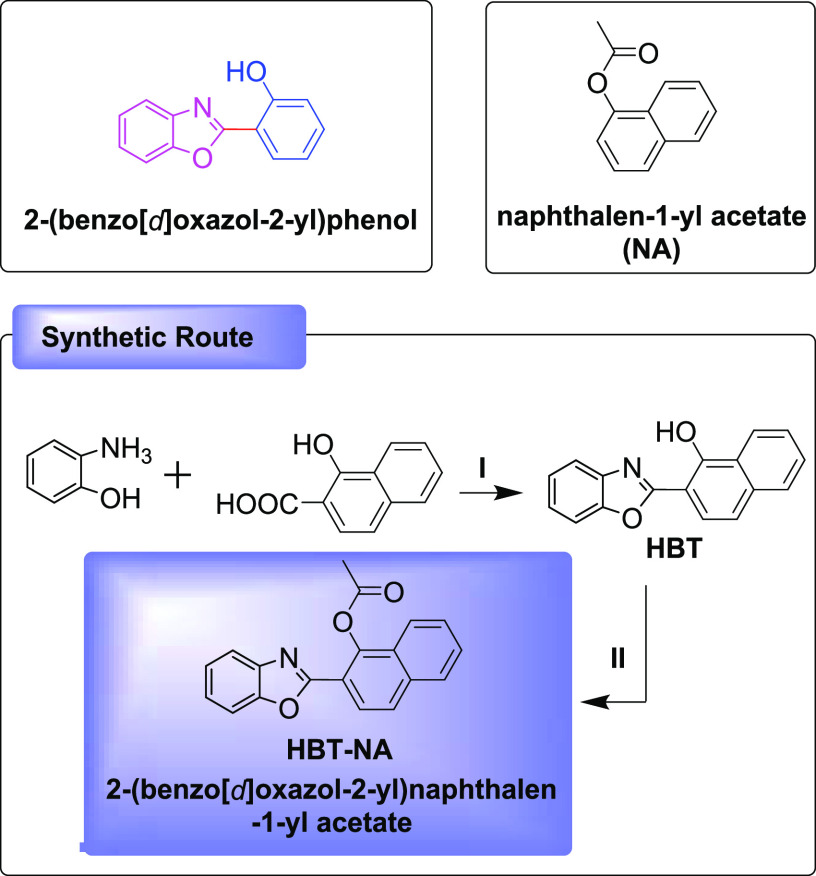
Molecular Structure and Synthetic Route for **HBT-NA**

The synthetic methods and routes to **HBT-NA** are given in Scheme 1. During the synthesis, thin-layer chromatography (TLC) was used to monitor in real time the formation of the intermediates and **HBT-NA**. The separation and purification of the products were achieved using column chromatography (silica gel, 200–300 mesh). **HBT-NA** and intermediates were characterized using an LC-ESI-qTOF mass spectrometer and 400 or 600 MHz NMR spectrometers. The fluorescence spectra of **HBT-NA** were obtained using a fluoromax-4 spectrophotometer (HORIBA-PLUS-C). The ultraviolet absorption spectra of **HBT-NA** were measured using a Cintra 2020 spectrophotometer (GBC Australia).

### Synthesis of 2-(Benzo[*d*]oxazol-2-yl)naphthalen-1-ol (Intermediate Product, **HBT**)

2-Aminophenol (2.0 mmol, 220 mg) and 1-hydroxy-2-naphthoic acid (2.0 mmol, 376 mg) were mixed in methylbenzene (20 mL) and heated to 80 °C with stirring for 1.0 h. Then, PCl_3_ (2.4 mmol, 324 mg) was added dropwise into the mixture and held at 40 °C. After the PCl_3_ was added, the mixture was then heated to reflux for 6.0 h and monitored using TLC. The crude product **HBT** was obtained after the solvent was removed under reduced pressure at the end of the reaction. **HBT** was purified using silica gel column chromatography, with DCM/ethyl acetate (100:1–10:1, v/v) as an eluent. **HBT** (2-(benzo[*d*]oxazol-2-yl)naphthalen-1-ol) was obtained as a yellow solid (228 mg). Yield: 87%. ^1^H NMR (600 MHz, DMSO-*d*_6_) δ: 13.47 (s, 1H), 8.39 (d, *J* = 8.2 Hz, 1H), 8.22 (d, *J* = 8.0 Hz, 1H), 8.14 (d, *J* = 8.1 Hz, 1H), 7.95 (d, *J* = 8.0 Hz, 1H), 7.88 (d, *J* = 8.6 Hz, 1H), 7.67 (t, *J* = 7.4 Hz, 1H), 7.62 (dd, *J* = 15.2, 8.1 Hz, 2H), 7.57 (d, *J* = 8.7 Hz, 1H), 7.51 (t, *J* = 7.6 Hz, 1H); ^13^C NMR (151 MHz, DMSO-*d*_6_) δ: 169.37, 154.96, 151.61, 135.68, 132.84, 129.25, 128.31, 127.60, 126.83, 126.11, 125.01, 124.88, 123.67, 122.87, 122.19, 120.26, 110.59. HRMS: *m*/*z* calcd for C_17_H_11_NO_2_ + H^+^: 262.0868, found, 262.0864.

### Synthesis of 2-(Benzo[*d*]oxazol-2-yl)naphthalen-1-yl Acetate (Product, **HBT-NA**)

**HBT** (0.36 mmol, 180 mg) and triethylamine (0.43 mmol, 43 mg) were dissolved in dichloromethane and stirred under N_2_ for 10 min. Then, acetyl chloride (0.42 mmol, 33 mg) was dissolved into dichloromethane and was added dropwise to the mixture at 0 °C. After addition, the mixture was stirred for 2.0 h at room temperature and monitored using TLC. When the reaction was complete, 80 mL of water was added to the mixture to quench the reaction. The mixture was separated, and the organic phase was collected. Crude **HBT-NA** was obtained on the removal of the solvent under reduced pressure. **HBT-NA** was then purified using silica gel chromatography with petroleum ether/ethyl acetate (100:1 to 30:1, v/v) as an eluent. **HBT-NA** (2-(benzo[*d*]oxazol-2-yl)naphthalen-1-yl acetate) was obtained as a white solid (236 mg). Yield: 78%. ^1^H NMR (600 MHz, DD) δ: 8.38 (dd, *J* = 8.6, 4.4 Hz, 1H), 8.13 (d, *J* = 8.1 Hz, 1H), 7.94 (d, *J* = 7.9 Hz, 1H), 7.93–7.88 (m, 2H), 7.86 (d, *J* = 8.7 Hz, 1H), 7.62–7.56 (m, 2H), 7.55–7.50 (m, 1H), 7.46–7.38 (m, 1H), 2.65 (s, 3H). ^13^C NMR (151 MHz, CDCl_3_) δ: 169.17, 162.85, 152.96, 145.23, 135.38, 135.27, 128.11, 127.94, 127.39, 127.36, 126.60, 126.44, 125.95, 125.50, 123.41, 122.52, 122.39, 121.39, 21.70. HRMS: *m*/*z* calcd for C_19_H_13_NO_3_ + H^+^: 304.0974, found, 304.0977.

### Monitoring the Structural Changes of **HBT-NA** Using ^1^H NMR

The products formed during the reaction of **HBT-NA** with **α-NAE**/**ANAE** were purified as follows: (1) the mixture was separated using an ultrafiltration tube, and the filtrate with a molecular weight below 1000 was obtained; (2) the filtrate was freeze-dried; and (3) then purified using silica gel column chromatography with DCM/ethyl acetate as an eluent. Changes of **HBT-NA** during the reaction process were monitored using high-performance liquid-liquid high-resolution mass spectrometry. **HBT-NA** (5.0 mM) and the corresponding equivalent ratio of **α-NAE** or **ANAE** in PBS were used for all of the reactions.

### Flow Cytometry for Typing of Lymphocytes and Analysis of Immunocompetence

Pure and highly immunoreactive T lymphocytes, B lymphocytes, and nontyped lymphocytes were used in this work, which were obtained from mice. The pure and highly immunoreactive T lymphocytes and B lymphocytes were used as control groups and were incubated with **HBT-NA** (2.0 μmol) for 30 min, where the excitation wavelength for the blue channel was 352 nm, the detection wavelength for the blue channel was 390–440 nm, the excitation wavelength for the green channel was 413 nm, and detection wavelength for the green channel was 500–560 nm. The threshold value was set by the fluorescence intensity of the control group (pure and highly immunoreactive T lymphocyte group and the pure and highly immunoreactive B lymphocyte group). The immunocompetence was analyzed using the activity of **α-NAE** and **ANAE**.

## Results and Discussion

### Molecular Design for the Differential Detection of **α-NAE** and **ANAE**

We designed a dual-factor synergistically regulated ESIPT-based probe (**HBT-NA**) for the simultaneous and differential monitoring of changes in the catalytic hydrolytic function of **α-NAE** and **ANAE** using the high-fidelity output signal. The selectivity, sensitivity, and dual-factor synergistic activation of the probe for the catalytic hydrolytic function of **α-NAE** and **ANAE** are key points for the molecular design. First, 2-(benzo[*d*]oxazol-2-yl)phenol (**HBT**, Scheme 1) was selected as the platform due to the efficient four-level photochemical capability, which is advantageous to improve the sensitivity and response speed. More importantly, the molecular design platform is more susceptible to proton transfer under neutral conditions, which is key to realizing the differential monitoring of changes in the catalytic hydrolytic function of **α-NAE** and **ANAE**, as part of the two factors required to activate the recognition output signals, *i.e*., appropriate pH conditions. To achieve high selectivity for nonspecific esterases, the naphthalen-1-yl acetate (**NA**, Scheme 1) was added to the probe as the specific activation group. This is the second of the two factors required to activate the recognition output signals, *i.e*., enzymatic activity. Based on this design strategy, *i.e*., differential regulation of the ESIPT process using two-factors, we anticipated that **HBT-NA** could simultaneously monitor in real time the catalytic hydrolytic function of **α-NAE** and **ANAE** using differential output signals. The high-fidelity differential output signals could then be used to precisely type T lymphocytes and B lymphocytes among nontyped lymphocytes and simultaneously evaluate their immunocompetence. The molecular structure and properties of **HBT-NA** and intermediate products are given in Scheme 1 (See Supporting Information for characterization data).

### Spectral Changes of HBT-NA Toward α-NAE and ANAE

**Figure 1:**
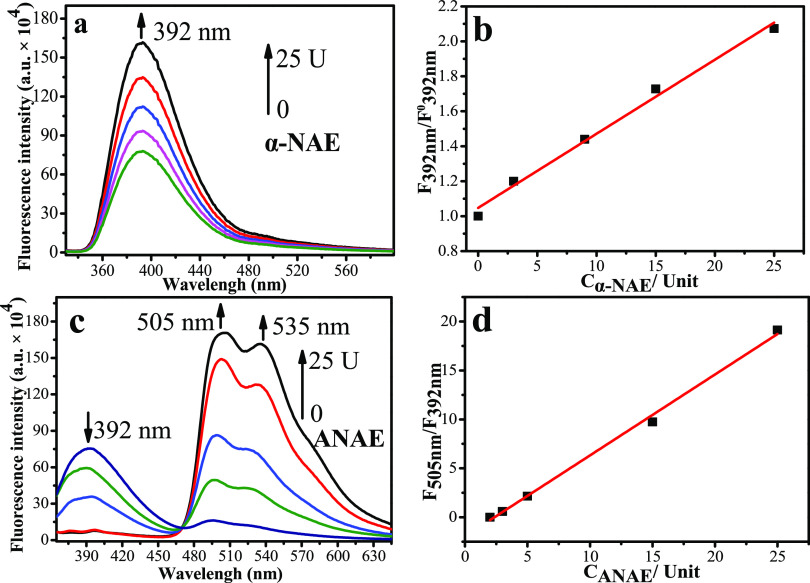
Spectral data of **HBT-NA** (5.0 μM). (a). Emission spectra of **HBT-NA** with **α-NAE** (0–25 U) in PBS buffer (pH = 7.4). (b). Linear relationship between **HBT-NA** and **α-NAE** (0–25 U) in PBS buffer solution (pH = 7.4); the detection limit of **HBT-NA** for **α-NAE** is 0.1621 U. (c). Emission spectra of **HBT-NA** for **ANAE** (0–25 U) in PBS buffer solutions (pH = 6.0). (d). Linear relationship between **HBT-NA** and **ANAE** (0–25 U) in PBS buffer solution (pH = 6.0); the detection limit of **HBT-NA** for **ANAE** is 0.09364U.

The spectral response including the absorption spectra and emission spectra of **HBT-NA** for **α-NAE** and **ANAE** were investigated using buffers with different pH values. The absorption spectra (Figure S1a) and the optical data (Table S1) indicated that **HBT-NA** (5.0 μM, ε = 10655 M^–1^ cm^–1^) exhibited an absorption peak at 320 nm at pH 7.4. With increasing **α-NAE**, the absorption peak does not change, but its intensity increases slightly in PBS buffer (pH = 7.4, Figure S1a), while there is a significant fluorescence enhancement in the emission spectra for **α-NAE** (Figure 1a). In the absence of **α-NAE**, **HBT-NA** was weakly fluorescent (F0_392 nm_, Φ°_**HBT-NA**_ = 0.13, λ_em-max_ = 392 nm, Table S1) in PBS buffer solutions (pH = 7.4, Figure 1a). When **HBT-NA** reacted with **α-NAE**, the fluorescence intensity was significantly enhanced with increasing **α-NAE** (0–25 U) at 392 nm at neutral pH (PBS buffer solutions, pH = 7.4) over a very short time (approximately 60 s, Figure S1b). The fluorescence quantum yield increases to 0.27 when the activity of **α-NAE** is increased to 25 U (Φ_**HBT-NA**_^25U^/Φ_**HBT-NA**_^0^ = 2.1) and then plateaus. Furthermore, the fluorescence enhancement of **HBT-NA** for **α-NAE** (F_392 nm_/F_392 nm_^0^) exhibited a good linear relationship (F_392 nm_/F_392 nm_^0^ = 0.042 C_**α-NAE**_ + 1.1, *R*^2^ = 0.99) with the activity of **α-NAE** (0–25 U, Figure 1b), producing a *v*_max_ of 3.750 μmol/L·S (Figure S1c). However, for **ANAE** at neural pH, no absorption or emission spectral changes of **HBT-NA** were observed (Figure S1d) even after 2 h. This is because **ANAE** was not active at neutral pH.

However, **ANAE** in acid solutions (PBS buffer, pH = 6.0) can cause significant changes of **HBT-NA** within 2 min in the absorption and emission spectra. With increasing **ANAE** to 25 U, the absorption peak was red-shifted from 320 nm to 370 nm and 400 nm in PBS buffer solution (pH = 6.0, Figure S1e), While the fluorescence spectra exhibited significant changes toward **ANAE** (Figure 1c). In the absence of **ANAE**, **HBT-NA** emitted a weak fluorescence (Φ_**HBT-NA**_ = 0.11, λ_em-max_= 392 nm) in PBS buffer solution (pH = 6.0). However, when **HBT-NA** reacts with **ANAE**, a strong fluorescence at 505 and 535 nm (pH = 6.0) was observed (Figure 1c), and the intensity increased with increasing **ANAE** (0–50 U). A good linear relationship (F_505 nm_/F_392 nm_ = 0.83C_**ANAE**_ – 1.95, *R*^2^ = 0.99, Figure 1d) was obtained between **ANAE** (0–25 U) and the fluorescence intensity ratio at 505 and 392 nm (F_505 nm_/F_392 nm_). Significantly, these differential spectral changes of **HBT-NA** toward **α-NAE** and **ANAE** are complete within 50 s and then plateau (Figure S1f), which is extremely conducive for the real-time differential monitoring of **ANAE**. The *v*_max_ with ANAE was determined to be 6.124 μmol/L·S (Figure S1g).

**Figure 2:**
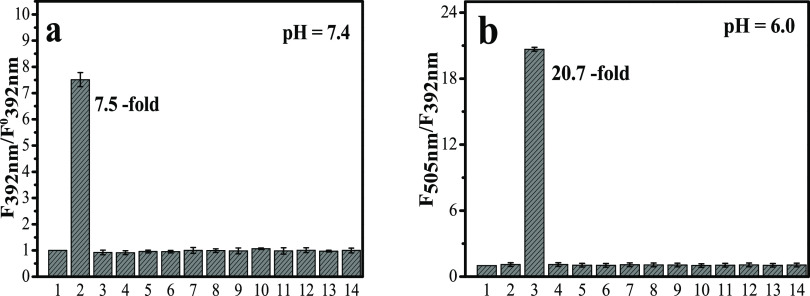
Selectivity experiments (a, pH = 7.4) and (b, pH = 6.0); 1, control; 2, **α-NAE**; 3, **ANAE**; 4, cholinesterase; 5, alkaline phosphatase; 6, nuclease; 7, phospholipase; 8, sulfatase; 9, sphingomyelinase; 10, hepatic lipase; 11, endothelial lipase; 12, lipoprotein lipase; 13, lysosomal acid lipase; and 14, acid cholesteryl ester hydrolase. Excitation wavelength = 320 nm. **HBT-NA**: 5.0 μM. Data were obtained from replicate experiments (*n* = 5).

Subsequently, the selectivity of **HBT-NA** for **α-NAE** and **ANAE** was evaluated. As shown in Figure 2a (pH = 7.4) and Figure 2b (pH = 6.0), there were no changes observed for 11 kinds of lipases (cholinesterase, alkaline phosphatase, nuclease, phospholipase, sulfatase, sphingomyelinase, hepatic lipase, endothelial lipase, lipoprotein lipase, lysosomal acid lipase, acid cholesteryl ester hydrolase) at different pH values, and similar results were obtained using 13 kinds of ions and 13 kinds of bioactive small molecules (Figure S2). These results indicated that the monitoring ability of **HBT-NA** for **α-NAE** and **ANAE** was highly specific.

### Mechanism of Spectral Changes of **HBT-NA** with **α-NAE** and **ANAE**

**Figure 3:**
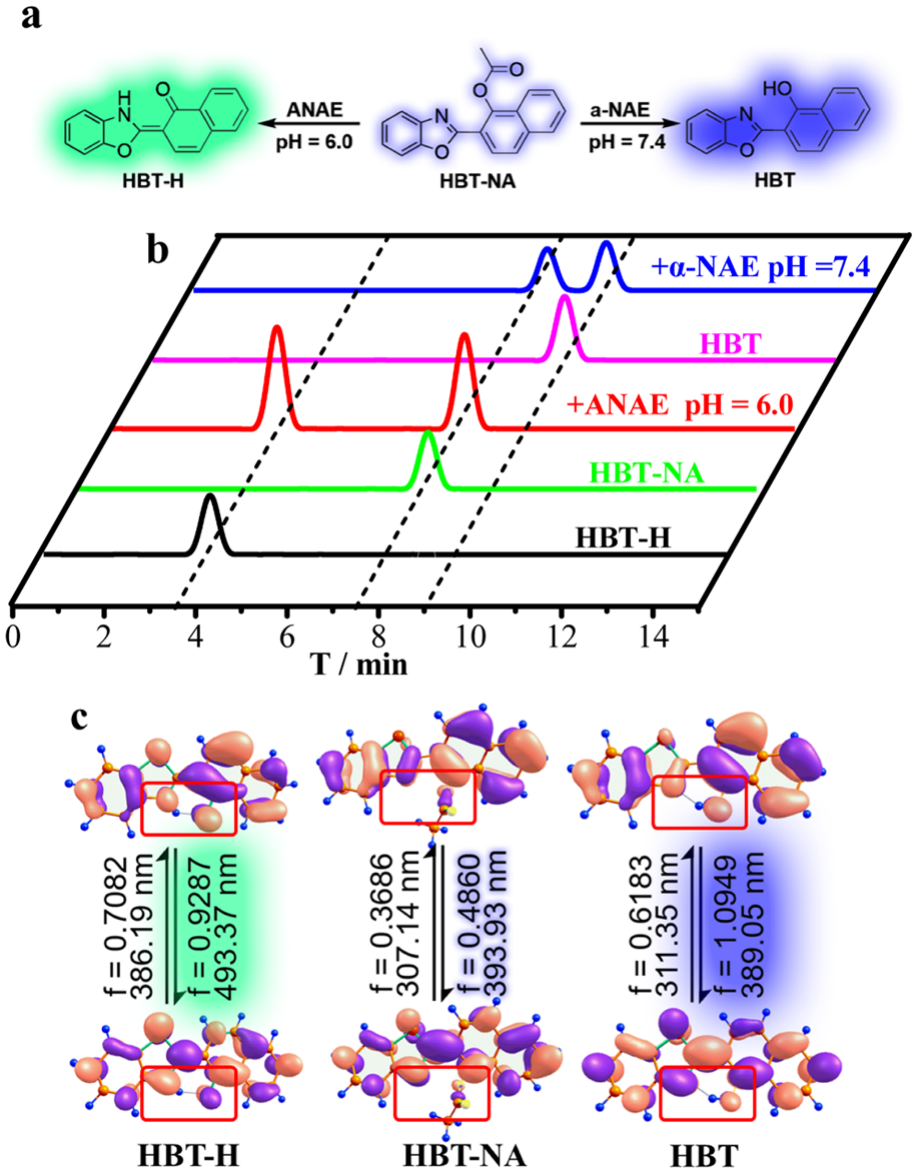
Structural changes of **HBT-NA** (a) and the HPLC results of **HBT-NA** under different conditions (b). Black line: pure **HBT-H**; green line: pure **HBT-NA**; purple line: pure **HBT**; red line: **HBT-NA** reacts with **ANAE** at pH = 6.0; blue line: **HBT-NA** reacts with **α-NAE** at pH = 7.4. (c) The orbital energy of HOMO and LUMO of **HBT-NA**, **HBT-H**, and **HBT** using Gaussian 16.

To explain the spectral changes (Figure 3a) of **HBT-NA** for **α-NAE** and **ANAE**, HPLC (Figure 3b) and Gaussian 16 (Figure 3c) were used to analyze the recognition process. The Gaussian 16 (Figure 3c) results indicated that **HBT-NA** exhibits maximum absorption and emission peaks at 307 and 393 nm, respectively, which were very close to the experimental results (Figures 1a and S1a). In pH = 7.4 PBS buffer, the chromatographic peak of **HBT-NA** (Mr = 303.0888) appeared at 7.75 min. When **HBT-NA** reacted with **α-NAE**, a new chromatographic peak at 9.05 min in pH = 7.4 PBS buffer was observed, which can be assigned to **HBT** (Mr = 261.0779) which is the enol (E) form (Figure 3a). The energy gap (Δ*E*) between the HOMO and LUMO of **HBT** is in line with that of **HBT-NA** (Figure 3c). But, the electron density of the oxygen of the hydroxyl increases (see the red box in Figure 3c); that is, the electron-donating ability increases. As such, the fluorescence intensity at 392 nm is enhanced, which belongs to the emission wavelength of the enol (E) form of **HBT**. Φ of **HBT** is twice that of Φ for **HBT-NA** (Table S1), which is consistent with the theoretical calculation and the spectral data in Figure 1. The spectroscopic data of **HBT** (Figure S1) and **HBT-NA** (Figure 1) indicated that there was only a change in intensity and no change in wavelength. In other words, the spectra indicated that an increased electron-donating ability leads to fluorescence enhancement. When **HBT-NA** reacted with **ANAE**, a new chromatographic peak at 3.66 min in pH = 6.0 PBS buffer was observed, which was assigned to **HBT-H** (Mr = 261.0796), which is the keto (K) form (Figure 3a). Gaussian 16 (Figure 3c) indicated that there is indeed an excited-state intramolecular proton transfer at pH = 6.0; thus, the absorption peak is at 386 nm and emission peak is at 493 nm and belongs to the emission wavelength of the keto (K) form of **HBT-H** (Figure 3a). The above experimental and theoretical calculations verified that the generation of differential signals during the recognition process is due to the generation of excited-state intramolecular proton transfer under the hydrolytic activity of enzymes and specific pH conditions (red boxes in Figure 3c).

### Monitoring the Intracellular Activity of **α-NAE** and **ANAE**

**Figure 4:**
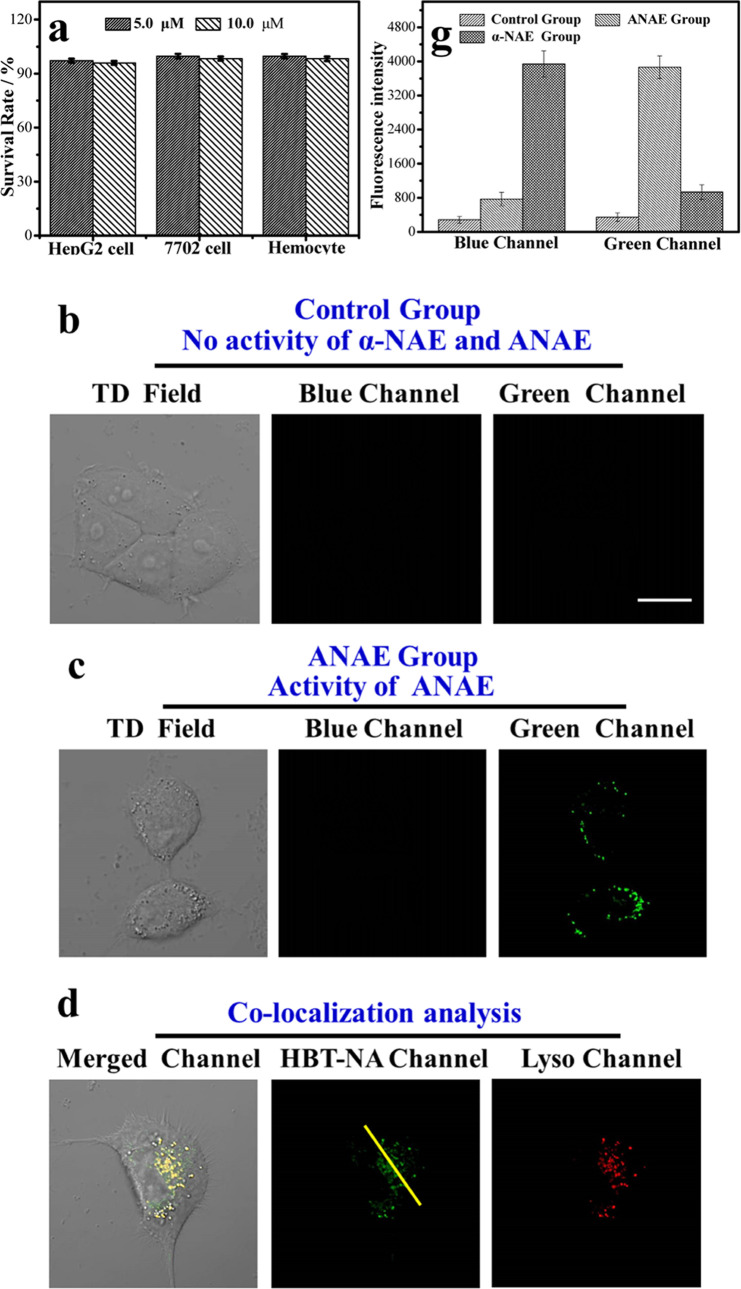

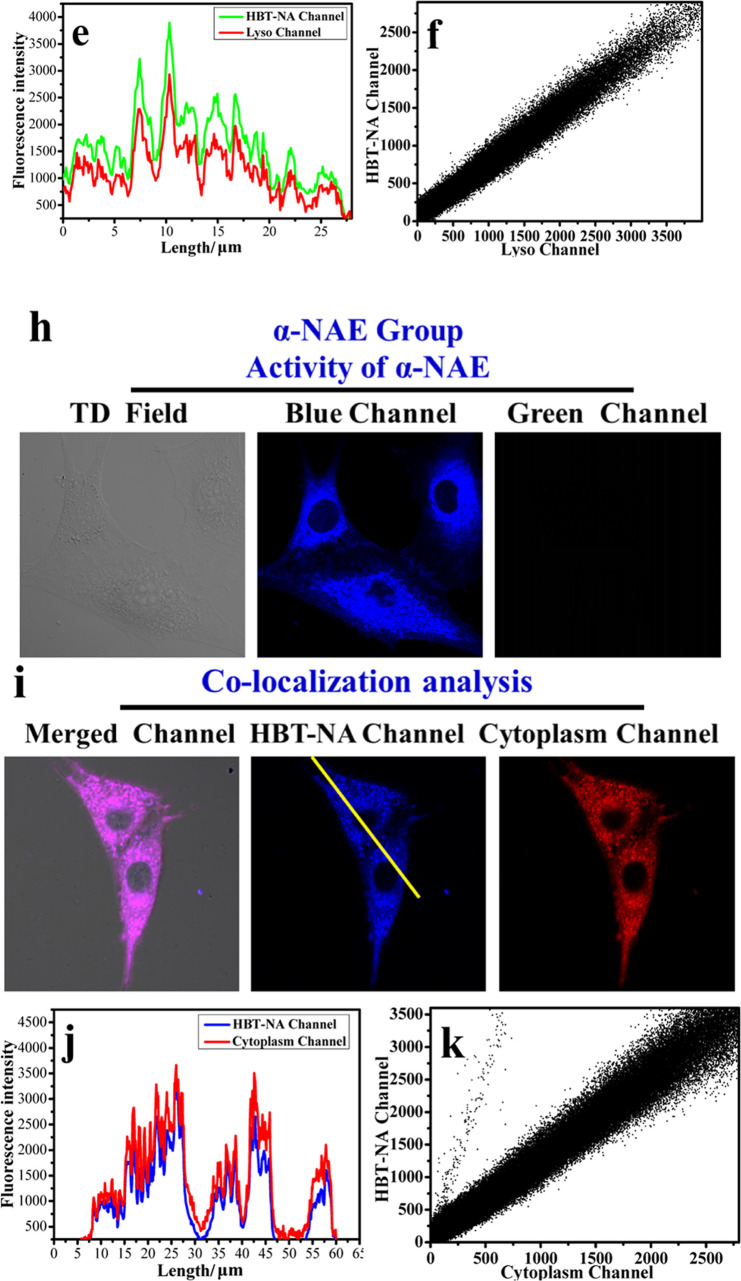
(a) Cell toxicity of **HBT-NA** (5.0 and 10.0 μM) for Hep G2 cells, 7702 cells, and hemocytes. (b) Control group: cell imaging of **HBT-NA** (5.0 μM**)** with no **α-NAE** and **ANAE** activity. The activities of **α-NAE** and **ANAE** were inhibited by NaF (1.0 mM). (c) **ANAE** group: cell imaging of **HBT-NA** (5.0 μM**)** under the activity of **ANAE**. (d) Colocalization experiments for **HBT-NA** (5.0 μM**)** with LysoTracker Red (1.0 μM) under the activity of **ANAE**. (e) Fluorescence intensity profile of the yellow line in **HBT-NA** channel and Lyso channel. (f) Intensity correlation plot of **HBT-NA** and LysoTracker Red in the same pixel between **the HBT-NA** channel and Lyso channel. (g) Fluorescence intensity of the blue channel and green channel in the control group, **ANAE** group, and **α-NAE** group. (h) **α-NAE** group: cell imaging of **HBT-NA** (5.0 μM**)** under the activity of **α-NAE**. (i) Colocalization experiments for **HBT-NA** (5.0 μM) and 5(6)-CFDA (1.0 μM) under the activity of **α-NAE**. (j) Fluorescence intensity profile of the yellow line in the **HBT-NA** channel and cytoplasm channel (5(6)-CFDA). (k) Intensity correlation plot of **HBT-NA** and 5(6)-CFDA in the same pixel between the **HBT-NA** channel and cytoplasm channel (5(6)-CFDA). Fluorescence collection wavelength for (b), (c), and (h): blue channel at 410–450 nm and green channel at 490–570 nm; excited at 405 nm. The fluorescence collection wavelength for (d): **HBT-NA** channel at 490–570 nm; excited at 405 nm; Lyso channel at 590–650 nm; excited at 559 nm. The fluorescence collection wavelength for (i): **HBT-NA** channel at 410–450 nm; excited at 405 nm; cytoplasm channel (5(6)-CFDA) at 550–600 nm; excited at 488 nm. Scale: 40 μm.

Encouraged by the excellent differential fluorescence signals of **HBT-NA** in aqueous media activated by the two factors, we evaluated the system in live cells. First, **HBT-NA** exhibited extremely low cell toxicity toward cancer cells (Hep G2 cells), normal cells (7702 cells), and hemocytes (Figure 4a). Furthermore, prior to the enzymatic activity analysis in living cells, the biocompatibility of **HBT-NA** (Figures S3–S6), including photostability, biological pH stability, and water solubility, were evaluated. **HBT-NA** exhibits low biotoxicity and excellent biocompatibility, making it convenient for monitoring intracellular enzyme activity. **HBT-NA** emits a very weak fluorescent signal in the blue channel (410–450 nm) and green channel (490–570 nm), which is almost negligible when the activities of **α-NAE** and **ANAE** are inhibited (Figure 4b). Even in an acidic environment, *i.e*., lysosome, negligible cellular fluorescence was observed (Figure 4b). That is to say, the environmental pH factor cannot activate differential fluorescence signals in the absence of enzyme activation. A bright fluorescence signal at 500–580 nm (green channel) was observed due to **ANAE** enzyme activity in the live cells (Figure 4c), whereas a bright fluorescence signal in the green channel was observed in the lysosome (Pearson coefficient = 97%) due to an acidic environment, where **ANAE** can exhibit enzymatic activity (Figure 4d4–4f). This is mainly due to the departure of the ester and proton transfer by the hydrolytic activity of **ANAE** under acid conditions. More importantly, the fluorescence intensity (F_green channel_) of the green channel gradually increases with increasing **ANAE** activity (Figure 4g). However, when there was only **α-NAE** in the live cells, only one bright fluorescence signal at 410–450 nm (blue channel, Figure 4h) was observed, and the fluorescence was only observed in the cytoplasm (Pearson coefficient = 92%) of the live cells (Figure 4i–4k) at neutral environment. This is because **α-NAE** only exhibits high enzyme activity under neutral conditions. That is, the hydrolytic activity of **α-NAE** can only function under these conditions. More importantly, the fluorescence intensity (*F*_blue channel_) of the blue channel gradually increased with the increasing enzyme activity of **α-NAE** (Figure 4g). These results indicate that **HBT-NA** can monitor the enzyme activity of **α-NAE** and **ANAE** in living cells using differential fluorescence signals.

### Typing lymphocytes and Evaluating Immunocompetence

**Figure 5:**
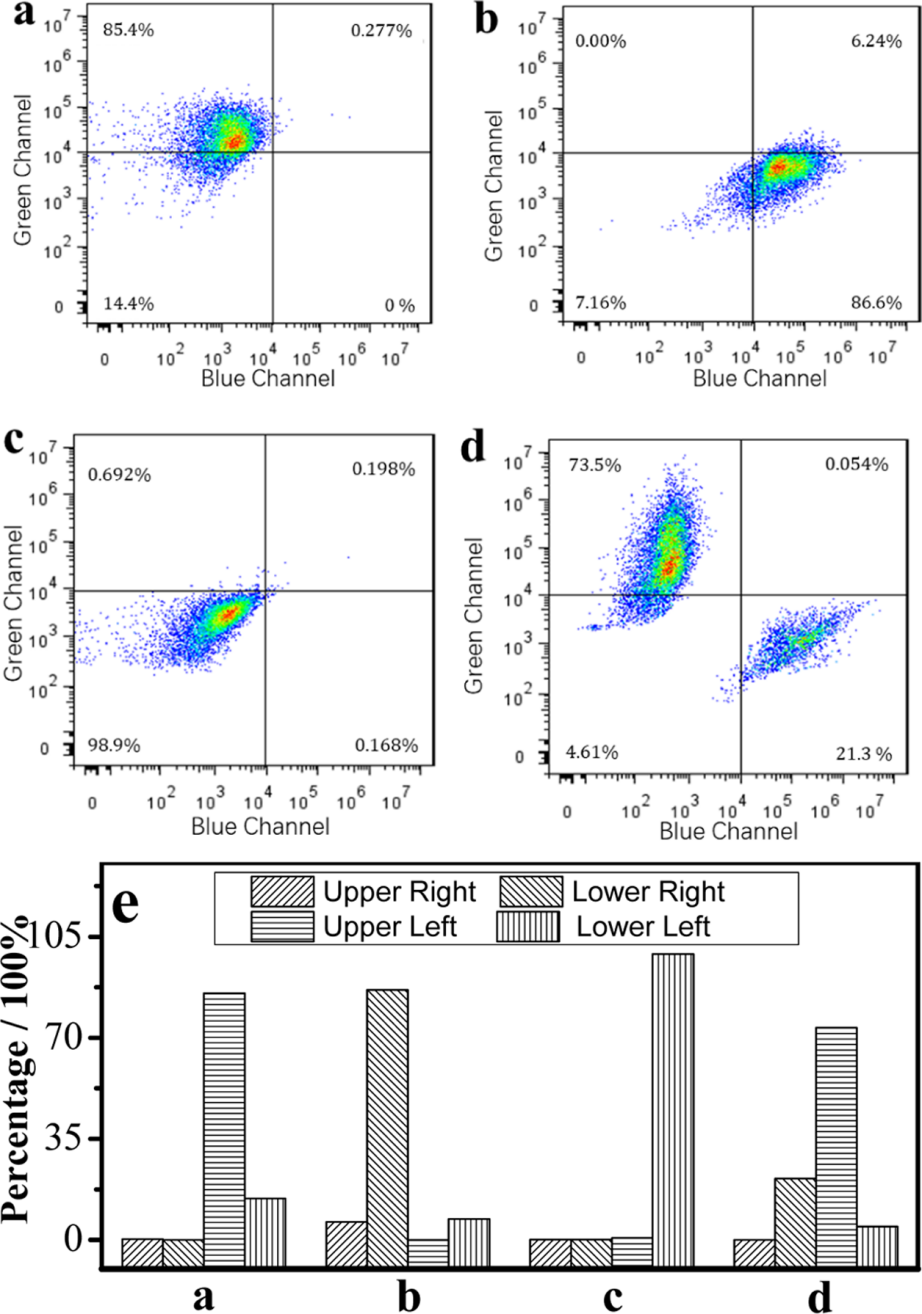
Flow cytometry for the typing of lymphocytes and the analysis of immunocompetence. (a) Pure and highly immunoreactive T lymphocyte; (b) pure and highly immunoreactive B lymphocyte; (c) lymphocytes in the primary stages of viral hepatitis; (d) lymphocytes in the advanced stages of viral hepatitis; and (e) quantitative data analysis. **HBT-NA**: 5.0 μmol. Blue channel: 392 nm, green channel: 505 nm.

The enzyme activities of **α-NAE** and **ANAE** in lymphocytes during immune response have become one of the breakthroughs in the study of immune diseases. The level of enzyme activity can type the kind of lymphocyte (*i.e*., T lymphocyte or B lymphocyte) and simultaneously reflect their immune activity. Thus, the precise typing of lymphocytes and the screening of immune cells using small changes of these two enzyme activities would be beneficial to help evaluate the immune function of living organisms. In this work, lymphocytes including T lymphocytes and B lymphocytes from different samples and viral hepatitis were stained using **HBT-NA** (5.0 μmol) and analyzed using flow cytometry (Figure 5).

**HBT-NA** (5.0 μmol) exhibited fluorescence signals with different intensities in the blue channel (392 nm) and green channel (505 nm) during the different stages of viral hepatitis. This was mainly attributed to the different activities of **α-NAE** and **ANAE** during these stages. As the activities of **α-NAE** and **ANAE** increase, the fluorescence signal intensities in the blue channel and green channel exceed 10^4^ au Therefore, this fluorescence intensity was used as a threshold to divide the quadrants. The pure and highly immunoreactive T lymphocytes and B lymphocytes isolated from mouse blood were used to help illustrate the thresholds (Figure 5a,5b). Figure 5a,5b, and 5e indicated that cells are mainly distributed in the upper left and lower right quadrants. The data in the two quadrants respectively are 85.4 and 86.3% for Figure 5a and 5b. For viral hepatitis samples obtained from mice Figure 5c–5e indicated that more T lymphocytes and B lymphocytes with high immune activity, respectively, enter the upper left and lower right quadrants as viral hepatitis progresses. The cell number in the upper left and lower right quadrants, respectively are 0.692 and 0.168% for the primary stage of viral hepatitis (Figure 5c,5e), while the number of cells in the upper left and lower right quadrants, respectively are 73.5 and 21.3% for advanced-stage viral hepatitis (Figure 5d,e). The above results are consistent with the results of the clinical standard staining methods (see Table S2), which indicated that the immunocompetence of lymphocytes gradually increased from the primary stage to the advanced stage during the progression of viral hepatitis. In addition, this method was much easier and more convenient than the standard clinical method. Thus, **HBT-NA** can be used as a potential tool for the typing of lymphocytes and the analysis of immunocompetence.

## Conclusions

The simultaneous and sensitive detection of nonspecific esterases, *i.e*., **α-NAE** and **ANAE** using a differential fluorescence signal by means of a dual-factor synergistically activated ESIPT-based probe (**HBT-NA**), has been achieved. The three key points in molecular design are selectivity, sensitivity, and dual-factor synergistic activation. With the molecular design, we set (1) catalytic hydrolytic function of **α-NAE** and **ANAE** and the appropriate pH conditions as the target for the molecular design; (2) 2-(benzo[*d*]oxazol -2-yl)phenol was the core of the fluorescent probe due to rapid response and different proton transfer under the effect of different pH; and (3) naphthalen-1-yl acetate was used as the specific reactive group for activation by the esterases. Based on this design strategy, **HBT-NA** emitted an absorption peak at 320 nm and weak fluorescence at 392 nm at pH 7.4. Significantly, **HBT-NA** generated different responses for **α-NAE** and **ANAE** under different environmental conditions. When **HBT-NA** reacted with **α-NAE** at pH = 7.4, the fluorescence intensity enhanced at 392 nm within approximately 60 s. However, when **HBT-NA** reacted with **ANAE**, ratiometric signals in the absorption and emission spectra were observed at pH = 6.0 within 2.0 min. Such differential fluorescence signals were used to detect the activity of **α-NAE** and **ANAE** in solutions and live cells. Importantly, based on the differential fluorescence signals, a highly sensitive method was developed to distinguish type T lymphocytes and B lymphocytes among nontyped lymphocytes using the enzyme activities of **α-NAE** and **ANAE**. More importantly, this method can be used in real time to evaluate the immune function of living organisms using flow cytometry in a rapid, sensitive, and quantitative fashion. Hence, **HBT-NA** could have potential applications in the ultrasensitive detection of the enzyme activity of **α-NAE** and **ANAE** suitable for real-time and precise typing of lymphocytes and monitoring of immunocompetence.

## Supporting Information Available

Procedures section, the synthesis of HBT-NA, the basic optical data and the absorption spectra of HBT-NA, the biocompatibility of HBT-NA, the clinical standard staining methods for the immunocompetence of lymphocyte, references, and attached spectra (PDF)
