# Supplementary material for: Dual-factor Synergistically Activated ESIPT-based Probe: Differential Fluorescence Signals to Simultaneously Detect α-Naphthyl Acetate and Acid α-Naphthyl Acetate Esterase
Source: Anal Chem. 2021 Oct 25;93(43):14471–80. doi: 10.1021/acs.analchem.1c02945 (PMC8728733; doi:10.1021/acs.analchem.1c02945)
Supplement: Supplementary file 1 — ac1c02945_si_001.pdf [file ac1c02945_si_001.pdf]

Supporting Information

Dual-factor Synergistically Activated ESIPT-based Probe: Differential Fluorescence Signals to Simultaneously Detect α-naphthyl Acetate and Acid α-naphthyl Acetate Esterase

Kui Wang,[a] Beidou Feng,[a] Yonggang Yang,[a] Yuehua Chen,[a] Yuzhu Wang,[b] Yafu Wang,[a] Lin Yang, [a,*] Kai Jiang,[a] Tony D James [a,c] and Hua Zhang [a,*]

[a] Key Laboratory of Green Chemical Media and Reactions, Ministry of Education; Henan Key Laboratory of Organic Functional Molecule and Drug Innovation; School of Chemistry and Chemical Engineering; Henan Normal University, Xinxiang, 453007, P.R. China.

[b] Department of Hepatobiliary and pancreatic surgery, Henan Provincial People's Hospital, Zhengzhou, 450003, P.R. China.

[c] Department of Chemistry, University of Bath, Bath, BA2 7AY, UK.

Corresponding Author's email: zhanghua1106@163.com, yanglin1819@163.com, Tel/Fax: +86-373-3326335.

Contents

1. Experimental procedures S-2
2. The synthesis of HBT-NA......S-5
3. The optical data and the absorption spectra of HBT-NA......S-6
4. Biocompatibility of HBT-NA......S-9
5. The clinical standard staining methods for the immunocompetence of lymphocytes......S-11
References......S-11
Spectra......S-12

1. Experimental procedures

Solvents and reagents of A.R. grade were used in all this research Column chromatograpy was used to purify compounds using silica gel (200-300 mesh). Cholinesterase, alkaline phosphatase, nuclease, phospholipase, sulfatase, sphingomyelinase, hepatic lipase, endothelial lipase, lipoprotein lipase, lysosomal acid lipase, acid cholesteryl ester hydrolase were obtained from Sigma Chemical Co. (USA). Doubly purified water was used in all experiments, which was prepared using by a Milli-Q system. Stock solutions of **HBT-NA** and intermediate products were used in spectrographic determination and cell experiments. NMR spectra were obtained using an Avance 400 or 600 MHz spectrometer (Bruker Co., Switzerland). Ultra-high-resolution electro-spray time-of-flight mass spectrometry (Compact) was used to measure the molecular mass. BD FACSCanto II (USA) was used to sort cells in flow cytometic analysis.

1.1 Spectrographic determination and quantum efficiency in vitro

A cintra 2020 spectrophotometer (GBC Australia) and the fluoromax-4 spectrophotometer (HORIBA-PLUS-C, USA) were used to measure absorption spectra and fluorescence spectra, respectively. In all spectral experiments, the final solutions contained < 5.0‰ DMSO. Each experiment was carried out in five replicates (n = 5). The relative fluorescence quantum yields were determined using Rhodamine B (Φ_F = 0.97 in methanol) by the following equation:

$$\Phi_x=\Phi_s (F_x/ F_s)(A_s/ A_x)(\lambda_{exs}/\lambda_{exx})(n_x/ n_s)^2 \qquad \text{(S1)}$$

where Φ represents quantum yield; F is the integrated area under the corrected emission spectrum; A is absorbance at the excitation wavelength; λ_{ex} is the excitation wavelength; n is the refractive index of the solution (because of the low concentrations of the solutions, 10^{-7}-10^{-8} mol/L, the refractive indices of the solutions were replaced with those of the solvents); and the subscripts x and s refer to the unknown and the standard, respectively. The detection limit was calculated by three times the standard deviation divided by the slope of the calibration curve. The data were obtained from replicate experiments (n = 5). T he Φ of **HBT-NA** (5.0 μM) for

α-NAE (25 U) and ANAE (25 U) in different pH solution (7.4 and 6.0) was made in accordance with the method (1.1 Spectrographic determination and quantum efficiency in vitro). α-NAE and ANAE were dissolved in different pH buffer solution, and than added **HBT-NA** (5.0 μM). To get accurate data, they reacted to each other for two minutes before taking measurements.

1.2 Photostability in solution

HBT-NA (5.0 μM) in PBS buffer (pH 7.4) at 25 °C. The solutions were irradiated by a 500W iodine-tungsten lamp situated 250 mm away for 7 h. An aqueous solution of sodium nitrite (50 g/L) was placed between the samples and the lamp as a light filter (to cut off the light shorter than 400 nm) and as a heat filter. The photostabilities were expressed in terms of remaining absorption (%) calculated from the changes of absorbance at the absorption maximum before and after irradiation by iodine-tungsten lamp. The absorbance was determined. The data were obtained from replicate experiments (n = 5).

1.3 Quantum Calculations

All the quantum chemical calculations were done with the Gaussian 16 suite. The geometry optimizations of the **HBT-NA** were performed using density functional theory (DFT) with Becke’s three- parameter hybrid exchange function with Lee-Yang-Parrgradient-corrected correlation functional (B3-LYP functional).

Gaussian 16 was used in quantum chemical, and the work of Han was as reference for setting up calculation parameter[S1]. The density functional theory (DFT)[S2] with B3-LYP and B3LYP-D3 functional were used in the geometry optimizations of the dyes. And 6-31G* basis set was utilized. No constraints to bonds/angles/dihedral angles were applied in the calculations and all atoms were free to optimize. The time-dependent density functional theory (TD-DFT)[S3, S4] at the B3LYP/6-31G** level was used to calculate electronic transition energies and corresponding oscillator strengths.

1.4 Cell Culture

HepG 2 cell lines, 7702 cell lines and the hemocyte were obtained from the Chinese Academy of Medical Sciences. The red-free Dulbecco's Modified Eagle's Medium (DMEM, WelGene) and eagle's minimum essential medium (MEM, WelGene) supplemented with penicillin/streptomycin and 10 % fetal bovine serum (FBS; Gibco) were used to culture cells in a CO_2 incubator at 37 °C. One day before imaging, the cells mentioned above were seeded into confocal dishes with well glass bottom (MatTek, 1# glass, 0.13-0.16 mm). They were incubated at 37 °C in 5.0 wt %/vol CO_2 for 24 h. And then, the cells were incubated with HBT-NA at a certain concentration.

1.5 Cytotoxicity

HepG 2 cell lines, 7702 cell lines and the hemocyte were prepared for cell viability studies in 96-well plates (1×10^5 cells per well that were incubated in 100 μL). The cells were incubated for an additional 24 h with dyes **HBT-NA** in different concentrations. Subsequently, 100 μL of 3-(4,5-dimethylthiazol-2-yl)-2,5-diphenyltetrazolium bromide (MTT, Sigma Chemical Co. U.S.A.) was added into each well, followed by further incubation for 4 h at 37 °C. The DMEM was removed and DMSO (200 μL/well) added to dissolve the reddish-blue crystals. Optical density (OD) was determined by a microplate reader (Spectra Max M5, Molecular Devices) at 570 nm with subtraction of the absorbance of the cell-free blank volume at 630 nm. The results from the six individual experiments were averaged. The relative cell viability (100%) was calculated using the following equation:

$$\text{Cell viability (\%)} = (OD_{dye}-OD_{k\text{-}dye})/(OD_{ctrl}-OD_{k\text{-}ctrl}) \times 100 \qquad \text{(S2)}$$

1.6 Flow cytometry

Kunming mouse (Eight weeks of age, Male) and Kunming mouse suffering from viral hepatitis (Eight weeks of age, Male) were purchased from National Laboratory Rodent Seed Center (Shanghai, China). Blood samples

were taken from their hearts, and then, T lymphocyte, B lymphocyte and lymphocyte were obtained by in vitro separation. All experiments were approved and followed the guidance of the Animal Ethics Committee, Xinxiang Medical University, Henan, China (Reference No. 2015016).

The pure and highly immunoreactive T lymphocyte, B lymphocyte and lymphocyte were incubated with **HBT-NA** (2.0 μM) for 30 min, then the cells were washed. The cells were then dispersed into PBS solution at level of 10,000 cells/500 μL. Samples were analyzed with the laser (352 nm and 413 nm) on a flow cytometer (BD FACSCanto II, USA). The average fluorescence intensity in 10,000 cells was obtained and analyzed with BD FACSDiva software. The excitation wavelength for the blue channel: 352 nm, blue channel: 390-440 nm; The excitation wavelength for the green channel: 413 nm, green channel: 500-560 nm.

2. The synthesis of HBT-NA

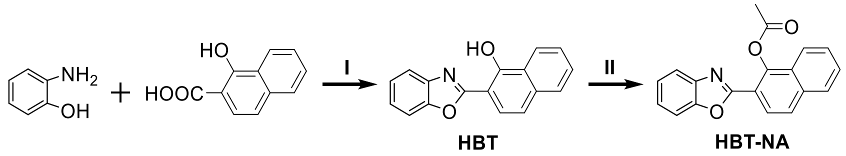

Synthesis of 2-(benzo[*d*]oxazol-2-yl)naphthalen-1-ol (intermediate product, HBT)

2-aminophenol (2.0 mmol, 220 mg), 1-hydroxy-2-naphthoic acid (2.0 mmol, 376 mg) were mixed in the solvent of methylbenzene (20 mL) and heated to 80 °C with stirring for 1.0 h. Then, PCl_3 (2.4 mmol, 324 mg) was added dropwise into the solvent and the reaction temperature was kept at 40 °C. After the PCl_3 was completely added, the mixture was heated to reflux for 6.0 h, and monitored by TLC. The crude product **HBT** was obtained after the solvent was removed under reduced pressure. Column chromatography on silica gel was used to purify **HBT**. And the eluent was DCM/Ethyl Acetate (100:1 to 10:1, v/v). **HBT** (2-(benzo[*d*]oxazol-2-yl)naphthalen-1-ol) was obtained as a yellow solid (228 mg, Yield 87%). ^{1}H NMR (600 MHz, DMSO-d6) δ: 13.47 (s, 1H), 8.39 (d, J = 8.2 Hz, 1H), 8.22 (d, J = 8.0 Hz, 1H), 8.14 (d, J = 8.1 Hz, 1H), 7.95 (d, J = 8.0 Hz, 1H), 7.88 (d, J = 8.6 Hz, 1H), 7.67 (t, J = 7.4 Hz, 1H), 7.62 (dd, J = 15.2, 8.1 Hz, 2H), 7.57 (d, J = 8.7 Hz, 1H), 7.51 (t, J = 7.6 Hz, 1H); ^{13}C NMR (151 MHz, DMSO-d6) δ: 169.37, 154.96, 151.61, 135.68, 132.84, 129.25, 128.31, 127.60, 126.83, 126.11, 125.01, 124.88, 123.67, 122.87, 122.19, 120.26, 110.59. HRMS: m/z calcd for $C_{17}H_{11}NO_2+H^+$: 262.0868, found: 262.0864.

Synthesis of 2-(benzo[*d*]oxazol-2-yl)naphthalen-1-yl acetate (product, HBT-NA)

The intermediate product (HBT, 0.36 mmol, 180 mg) and triethylamine (0.43 mmol, 43 mg) were dissolved in dichloromethane and stirred under N_2 for 10 min. Acetyl chloride (0.42 mmol, 33 mg) dissolved in dichloromethane and was added dropwise into the mixture at 0 ^{o}C. After complete addition, the solvent stirred for 2.0 h at room temperature, and monitored by TLC. When the reaction was complete, 80 mL of water was added into the mixture to quench the reaction. The mixture was separated by extraction using petroleum ether three times, and the organic phase was collected. Then, the crude product **HBT-NA** was obtained after the solvent was removed under reduced pressure. Column chromatography using silica gel was used to purify **HBT-NA** using petroleum ether/Ethyl Acetate (100:1 to 30:1, v/v) as eluent. **HBT-NA** (2-(benzo[*d*]oxazol-2-yl)naphthalen-1-yl acetate) was obtained as a white solid (236 mg,Yield 78%). ^{1}H NMR (600 MHz, DD) δ: 8.38 (dd, J = 8.6, 4.4 Hz, 1H), 8.13 (d, J = 8.1 Hz, 1H), 7.94 (d, J = 7.9 Hz, 1H), 7.93-7.88 (m, 2H), 7.86 (d, J = 8.7 Hz, 1H), 7.62-7.56 (m, 2H), 7.55-7.50 (m, 1H), 7.46-7.38 (m, 1H), 2.65 (s, 3H). ^{13}C NMR (151 MHz, $CDCl_3$) δ: 169.17, 162.85, 152.96, 145.23, 135.38, 135.27, 128.11, 127.94, 127.39, 127.36, 126.60, 126.44, 125.95, 125.50, 123.41, 122.52, 122.39, 121.39, 21.70. HRMS: m/z calcd for $C_{19}H_{13}NO_3+H^+$: 304.0974, found: 304.0977.

3. The optical data and the absorption spectra of HBT-NA

Table S1. The optical data of HBT-NA and its recognition derivatives

	HBT-NA	HBT-NA +α-NAE	HBT-NA +α-NAE	HBT	HBT-NA +ANAE	HBT-NA +ANAE	HBT-H
pH of solution	7.4	7.4	6.0	7.4	7.4	6.0	6.0
λ_{ex}/nm	320	321	320	325	320	320	322
ε / M^{-1} cm^{-1}	10655	18673	10576	19648	11067	22352	21564
λ_{em}/nm	392	392	392	392	392	392/505，535	505，535
Φ	0.13	0.27	0.12	0.28	0.13	--	--

ε Molar extinction coefficient;Φ fluorescence quantum yield, the measurements condition: the Φ of **HBT-NA** (5.0 μM) for **α-NAE** (25 U) and **ANAE** (25 U) in different pH solution (7.4 and 6.0) was made in accordance with the method (1.1 Spectrographic determination and quantum efficiency in vitro). **α-NAE** and **ANAE** were dissolved in different pH buffer solution, and than added **HBT-NA** (5.0 μM). To get accurate data, they were reacted with each other for two minutes before taking measurements.

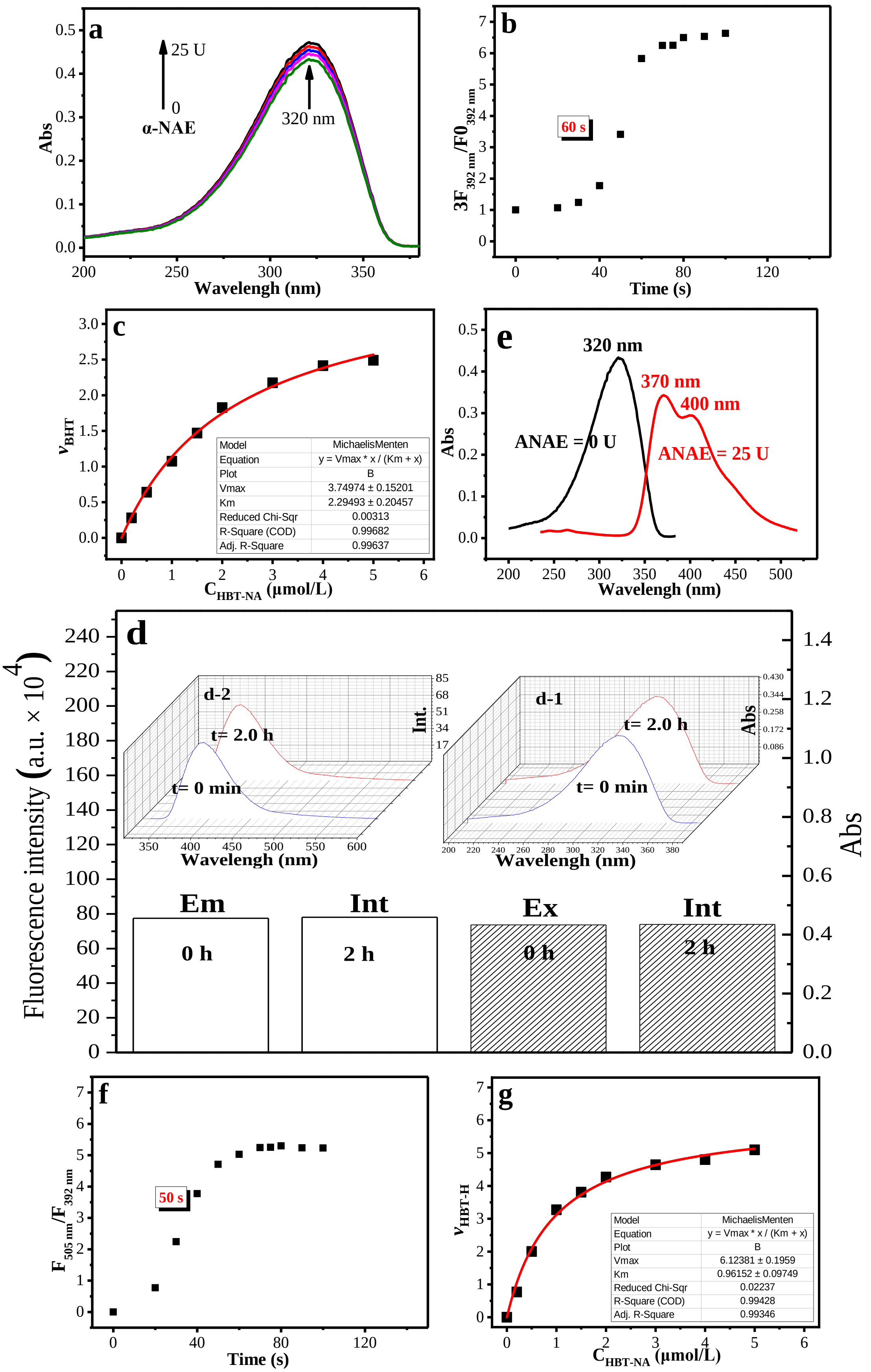
a
25 U
0
α-NAE
320 nm
Abs
Wavelengh (nm)
b
60 s
3F392 nm/F0392 nm
Time (s)
c
vBHT
CHBT-NA (μmol/L)
Model MichaelisMenten
Equation y = Vmax * x / (Km + x)
Plot B
Vmax 3.74974 ± 0.15201
Km 2.29493 ± 0.20457
Reduced Chi-Sqr 0.00313
R-Square (COD) 0.99682
Adj. R-Square 0.99637
e
320 nm
370 nm
400 nm
ANAE = 0 U
ANAE = 25 U
Abs
Wavelength (nm)
d
Fluorescence intensity (a.u. × 10^4)
Abs
d-2
t= 2.0 h
t= 0 min
Int.
Wavelength (nm)
d-1
t= 2.0 h
t= 0 min
Abs
Wavelength (nm)
Em
0 h
Int
2 h
Ex
0 h
Int
2 h
f
50 s
F505 nm/F392 nm
Time (s)
g
vHBT-H
CHBT-NA (μmol/L)
Model MichaelisMenten
Equation y = Vmax * x / (Km + x)
Plot B
Vmax 6.12381 ± 0.1959
Km 0.96152 ± 0.09749
Reduced Chi-Sqr 0.02237
R-Square (COD) 0.99428
Adj. R-Square 0.99346

Fig. S1. Spectral data of **HBT-NA** (5.0 μM). (a). The absorption spectrum changes of **HBT-NA** (5.0 μM) with **α-NAE** (0-25U) in PBS buffer solutions (pH = 7.4). (b). The reaction profile of **HBT-NA** (5.0 μM) with **α-NAE** (25U) in PBS buffer solutions (pH = 7.4). $3F_{392\ nm}/F^0_{392\ nm}$ is used as the ordinate to evaluate the fluorescence intensity change and to reduce the error of curve fitting. (c). The kinetic analysis of **α-NAE** (25U) enzymatic reaction using Michaels-Menten analysis. The equation is v= 3.750*s/(2.295+s). (d).The absorption and emission spectra changes of **HBT-NA** for **ANAE** in PBS buffer solutions (pH = 7.4). (e). The absorption spectra changes of **HBT-NA** with **ANAE** (0 U (black line) and 25 U (red line)) in PBS buffer solutions (pH = 6.0). (f). The reaction profile of **HBT-NA** with **ANAE** in PBS buffer solutions (pH = 6.0). (g). The kinetic analysis of **ANAE** (25U) enzymatic reaction using Michaels-Menten analysis. The equation is v= 6.124*s/(0.9615+s)

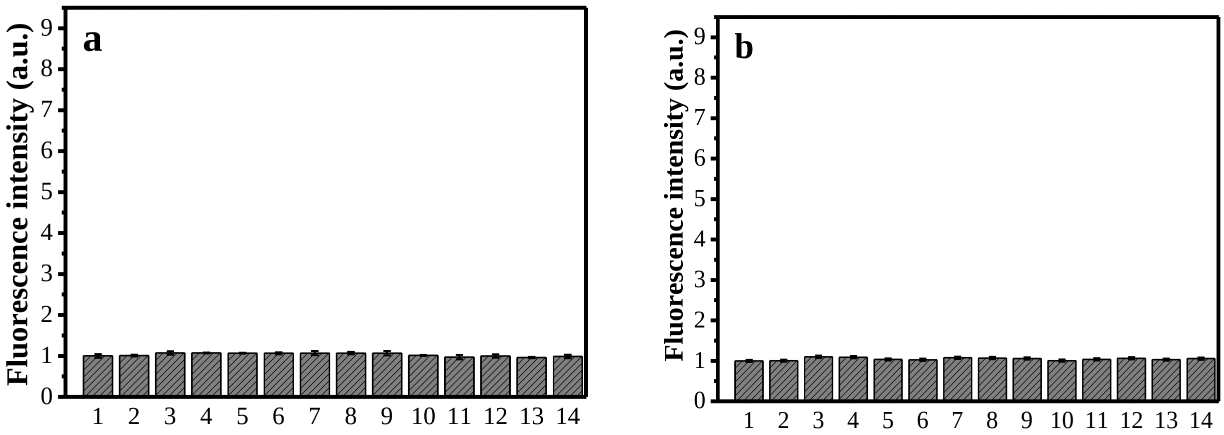

Fig. S2. (a) Selective experiment; 1, control; 2, arginine; 3, glycine; 4, aspartic acid; 5, 5-hydroxytryptamine; 6, catecholamine; 7, glutamic acid; 8, aminobutyric acid; 9, H_2S; 10, NO_2; 11, NO; 12, H_2O_2 ; 13, $ONOO^-$; and 14, $O_2^{\cdot-}$; (b) The influence of ions. 1. Control, 2. Ag^+ (0.10 mM), 3. Al^{3+} (0.10 mM), 4. NO_3^- (0.30 mM), 5. Ni^{2+} (0.10 mM), 6. Cl^- (0.20 mM), 7. Mg^{2+} (0.10 mM), 8. SO_4^{2-} (0.10 mM), 9. CO_3^{2-} (0.050 mM), 10. Na^+ (0.10 mM), 11. K^+ (0.10 mM), 12. Ca^{2+} (0.10 mM), 14. $H_2PO_4^-$ (0.10 mM). Excitation wavelength = 320 nm. emission wavelength = 392 nm and 505 nm. **HBT-NA**: 5.0 μM. Data were obtained from replicate experiments (n = 5)

4. Biocompatibility of HBT-NA

Prior to the cell assay, the biocompatibility of **HBT-NA** including photostability, biological pH stability, cell toxicity, water solubility and so on, were evaluated by fluorescence intensity as the evaluation parameter, respectively.

4.1 The photostability of HBT-NA under irradiation by iodine-tungsten lamp in PBS buffer

HBT-NA (5.0 μM) retained more than 98% of the fluorescence intensity in PBS buffer after they were continuously irradiated by an iodine-tungsten lamp for 6.0 h. The photostability of **HBT-NA** ensures that it can provide a stable response signal for the ultra-sensitive detection of the enzyme activity and the real-time precise typing of lymphocytes.

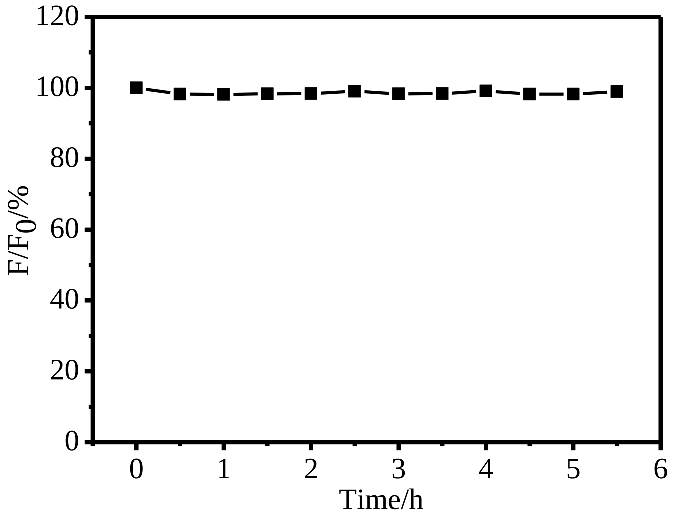

Fig. S3. The photostability of **HBT-NA** (5.0 μM) in PBS buffer (pH 7.4) at 25 °C. Iodine-tungsten lamp: 500W. Excitation wavelength = 320 nm. Emission wavelength = 392 nm

4.2 The biological photostability of HBT-NA in cells

HBT-NA (5.0 μM) retained more than 98.5 % of the fluorescence intensity in living cell after being continuously irradiated by a laser at 405 nm (initial power = 2.6 mM) for 6.0 h. Such biological photostability of **HBT-NA** ensures that it can provide a stable response signal for the ultra-sensitive detection of the enzyme activity and the real-time precise typing of lymphocyte.

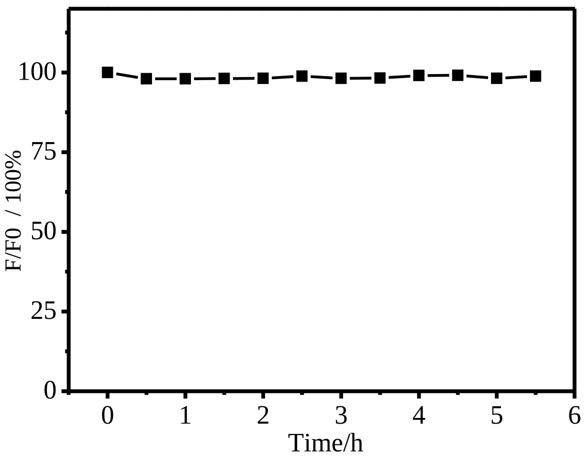

Fig. S4. The biological photostability of **HBT-NA** (5.0 μM) in cell. Excitation wavelength = 405 nm, signal collection wavelength: 500-550 nm

4.3 The pH-stability of HBT-NA

No any influence on the fluorescent signal was observed when the pH of phosphate buffer was varied from 3.0-11.0. Such kind of pH-stability of **HBT-NA** ensures that it can provide a stable response signal for the ultra-sensitive detection of the enzyme activities and the real-time precise typing of lymphocyte.

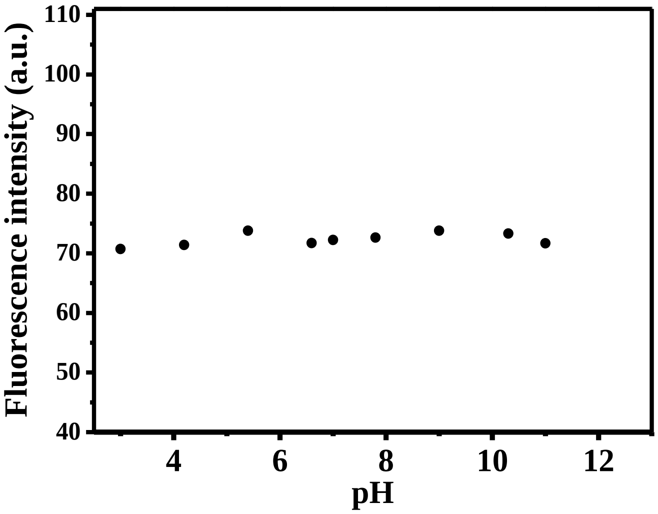

Fig. S5. The pH-stability of **HBT-NA** (5.0 μM) in pH range from 3.0 to 11.0. Excitation wavelength = 320nm. Emission wavelength = 392 nm

4.4 The water solubility of HBT-NA

The solubility of **HBT-NA** was 9.0 μM. The water solubility of **HBT-NA** ensures that it is suitable for for the ultra-sensitive detection of the enzyme activities and the real-time precise typing of lymphocytes.

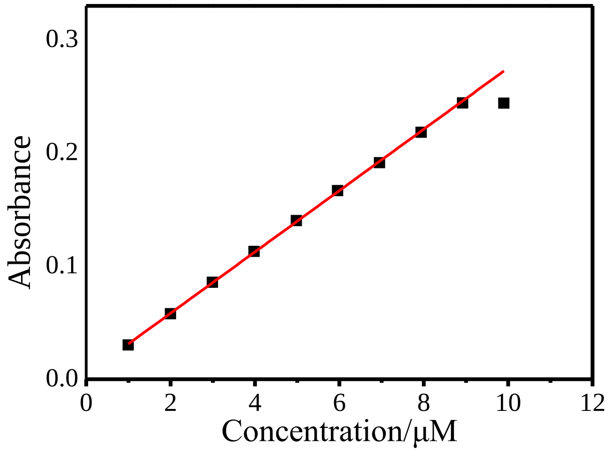

Fig. S6. The water solubility of **HBT-NA** in PBS buffer (pH 7.4) at 25 °C

5. The clinical standard staining methods for the immunocompetence of lymphocytes

Table S2. The immunocompetence of lymphocytes

	Low immunocompetence Lymphocyte	High immunocompetence T lymphocyte	High immunocompetence B lymphocyte
Primary stage of viral hepatitis	99.1	0.71	0.19
Advanced stage of viral hepatitis	5.4	72.9	21.7

References.

S1. Zhou, L.C., Zhao, G.J., Liu, J.F., Han, K.L., Wu, Y.K., Peng, X.J., Sun, M.T.. J. *Photochem. Photobiol. A: Chem*. **2007**, 187:305.

S2. Dreizler, M.R., Gross, U., Density, E.K., **1990**. Functional Theory. Springer Verlag, Heidelberg.

S3. Gross, E.K.U., Kohn, W. *Phys. Rev. Lett*. **1985**, 55:2850.

S4. Stratmann, R.E., Scuseria, G.E., Frisch, M.J. *J. Chem. Phys*. **1998**, 109:8218.

Attached Spectra.

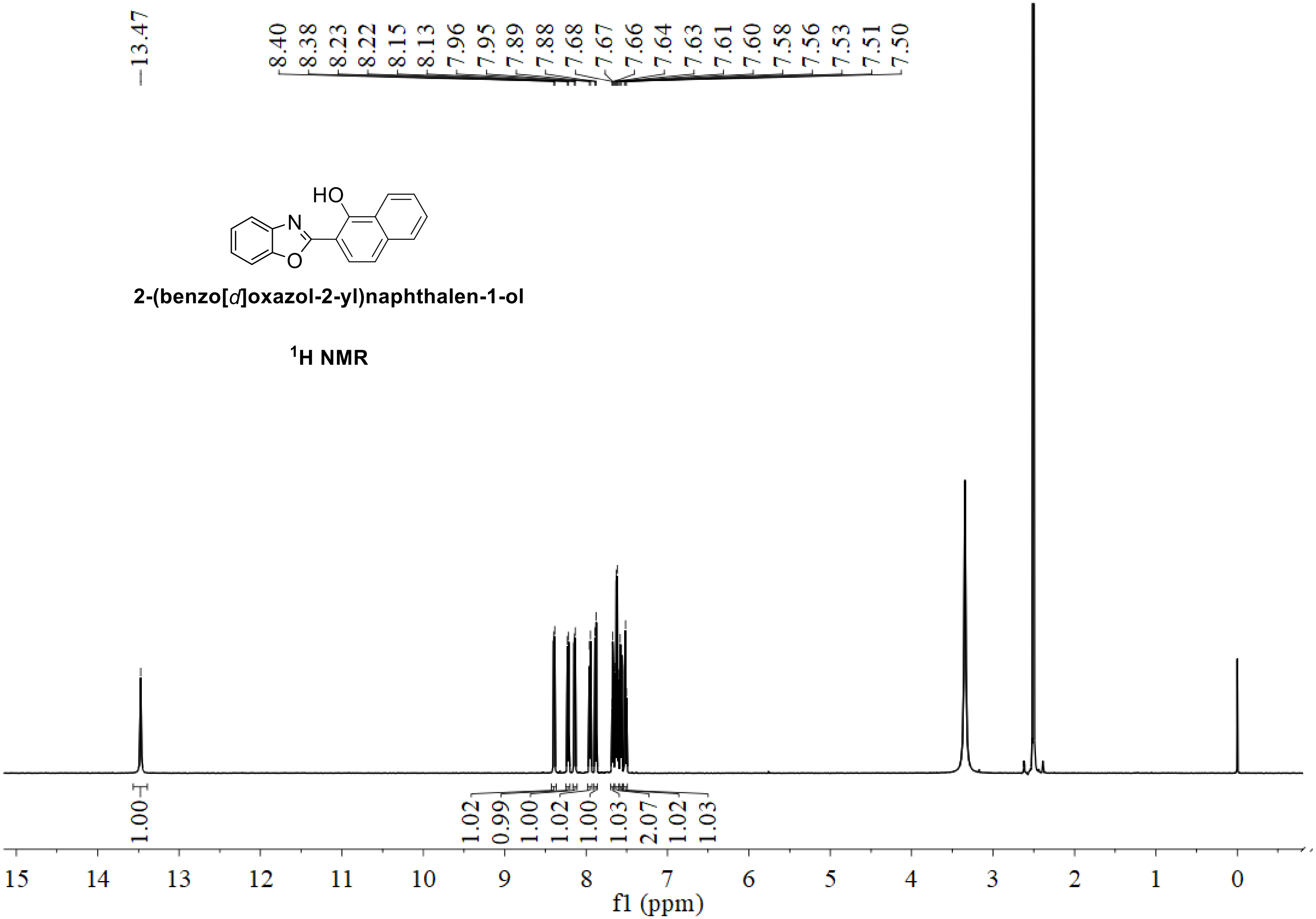

Fig. S7. The ^{1}H NMR of 2-(benzo[*d*]oxazol-2-yl)naphthalen-1-ol

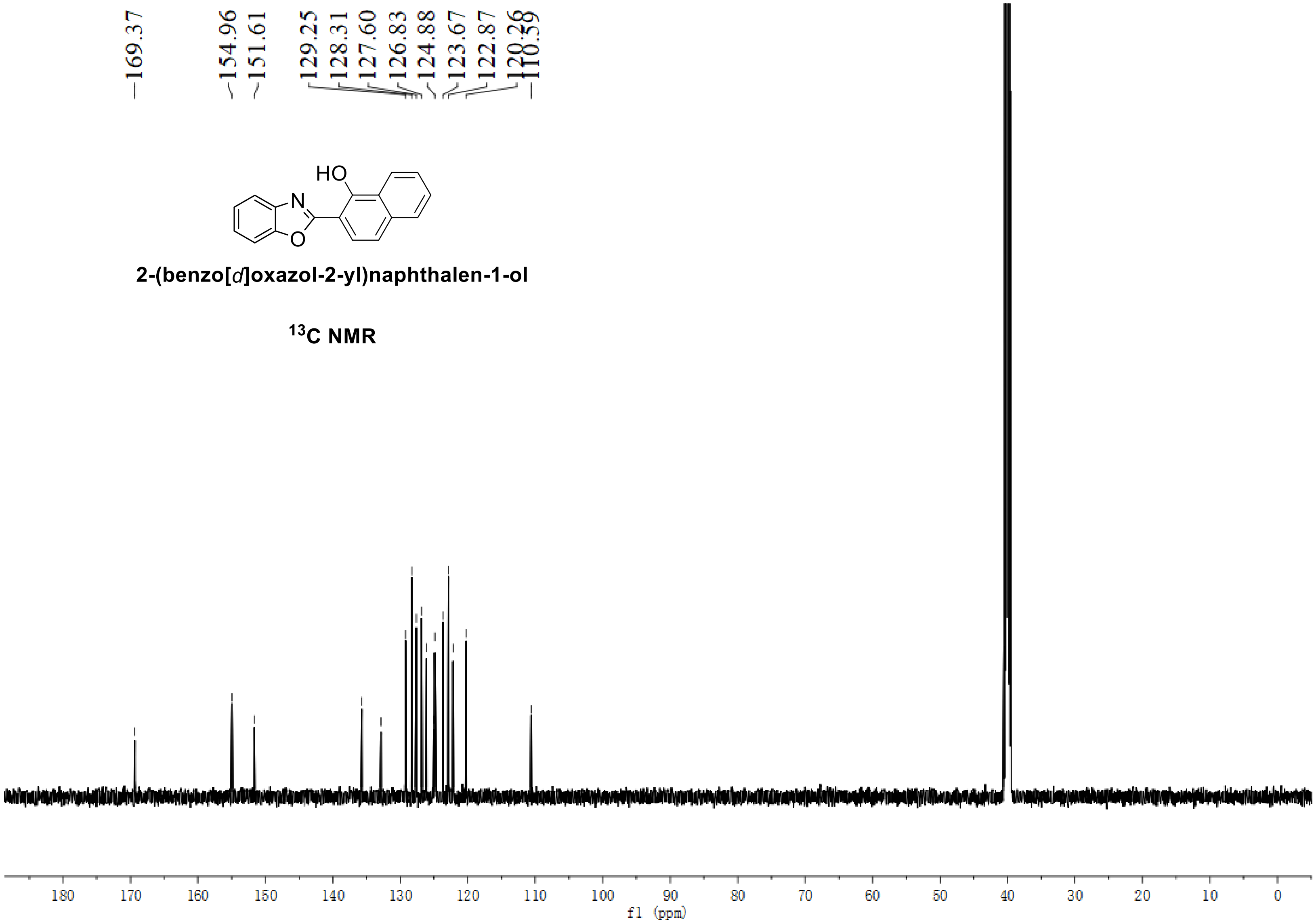

Fig. S8. The ^{13}C NMR of 2-(benzo[*d*]oxazol-2-yl)naphthalen-1-ol

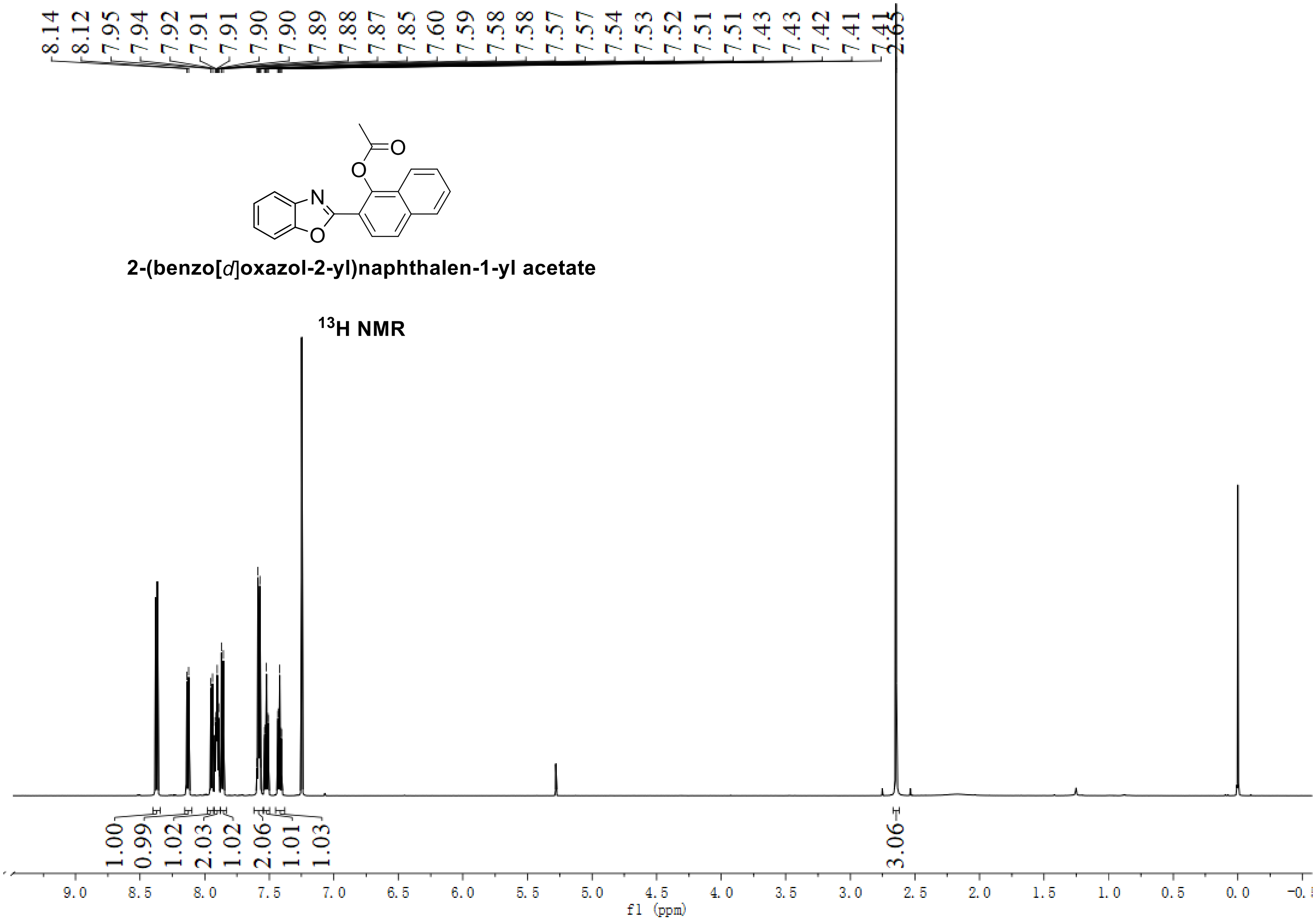

Fig. S9. The ^{1}H NMR of 2-(benzo[*d*]oxazol-2-yl)naphthalen-1-yl acetate

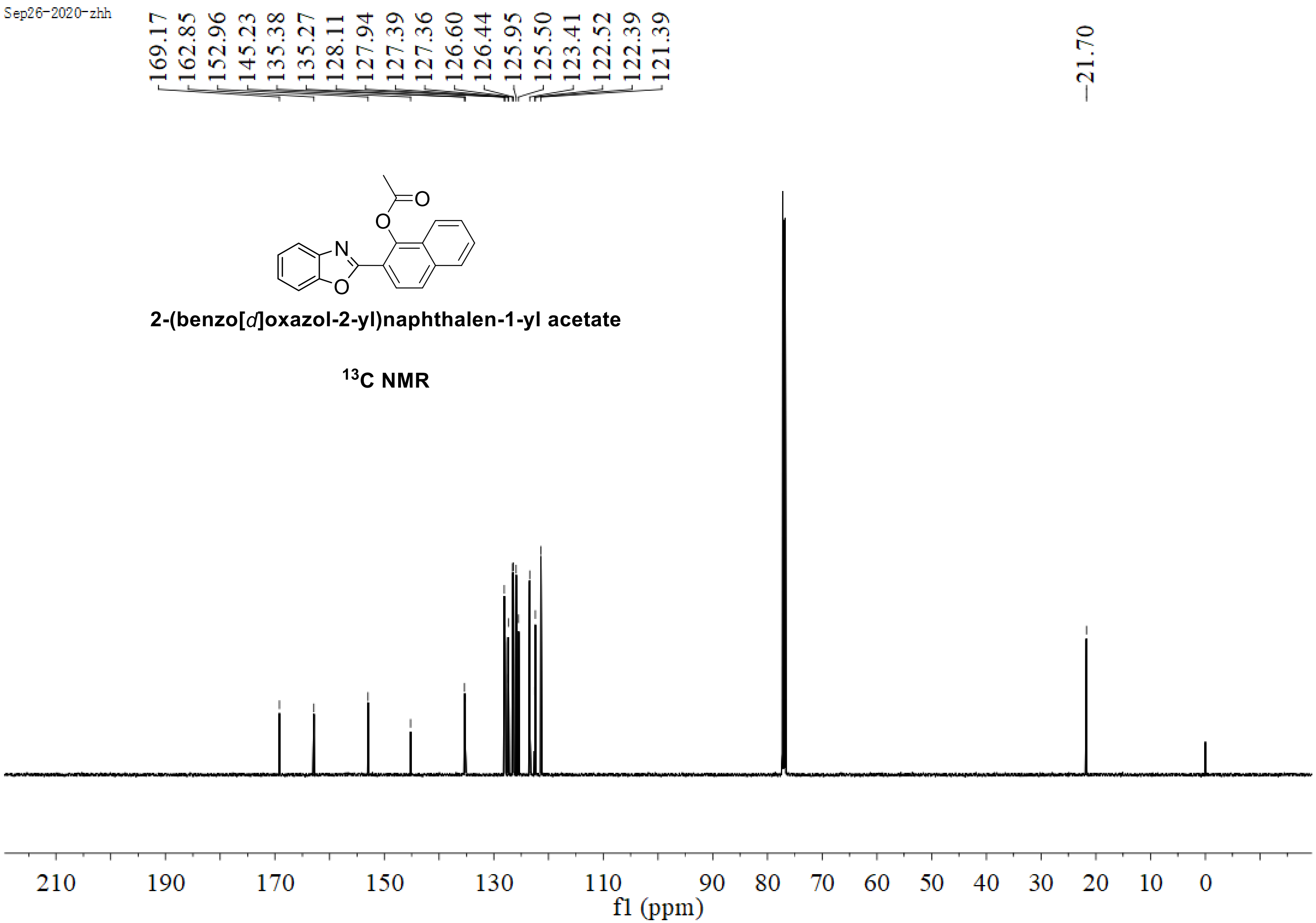

Fig. S10. The ^{13}C NMR of 2-(benzo[*d*]oxazol-2-yl)naphthalen-1-yl acetate
